# Demonstration of qubit operations below a rigorous fault tolerance threshold with gate set tomography

**DOI:** 10.1038/ncomms14485

**Published:** 2017-02-15

**Authors:** Robin Blume-Kohout, John King Gamble, Erik Nielsen, Kenneth Rudinger, Jonathan Mizrahi, Kevin Fortier, Peter Maunz

**Affiliations:** 1 Center for Computing Research, Sandia National Laboratories, Albuquerque, New Mexico 87185, USA; 2 Sandia National Laboratories, Albuquerque, New Mexico 87185, USA

## Abstract

Quantum information processors promise fast algorithms for problems inaccessible to classical computers. But since qubits are noisy and error-prone, they will depend on fault-tolerant quantum error correction (FTQEC) to compute reliably. Quantum error correction can protect against general noise if—and only if—the error in each physical qubit operation is smaller than a certain threshold. The threshold for general errors is quantified by their diamond norm. Until now, qubits have been assessed primarily by randomized benchmarking, which reports a different error rate that is not sensitive to all errors, and cannot be compared directly to diamond norm thresholds. Here we use gate set tomography to completely characterize operations on a trapped-Yb^+^-ion qubit and demonstrate with greater than 95% confidence that they satisfy a rigorous threshold for FTQEC (diamond norm ≤6.7 × 10^−4^).

## Abstract (web summary)

Quantum computation will depend on fault-tolerant error correction, which requires the chance for errors to occur to be below a certain threshold. Here the authors use gate set tomography as a means to rigorously characterize error rates of single-qubit operations of a qubit encoded in a trapped ion.

The field of quantum information processing has seen great growth over the past 30 years, driven by exciting quantum algorithms inaccessible to classical computers. Small quantum information processors have been demonstrated experimentally using superconducting circuits^1^,^2^,^3^, electrons in semiconductors^4^,^5^,^6^, trapped atoms and ions^7^,^8^,^9^ and photons^10^,^11^,^12^. Trapped ions are among the most reliable qubits available today; up to 14 qubits have been addressed in a single trap^13^, a 5-qubit quantum information processor has been realized^14^, and single-qubit gates have demonstrated randomized benchmarking (RB) infidelities as low as 10^−6^ (refs 15, 16, 17).

Unlike classical bits, qubits are intrinsically noisy and error-prone, and will require active, fault-tolerant quantum error correction (FTQEC^18^) to operate reliably. To function, FTQEC requires physical qubit operations to be high quality, with errors below a specific threshold. Fault tolerance (FT) thresholds for quantum computing have been proven against various noise models, and generally require per-gate failure rates between 10^−6^ and 10^−2^ (refs 19, 20, 21, 22). However, the particular metric of ‘error rate’ depends on the noise model. Against realistic general errors, including small unitary errors, thresholds are stated in terms of the gates’ diamond norm error, 
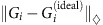
 (refs 20, 23, 24).

RB^25^,^26^, the most commonly used method for qubit characterization, measures a single error rate (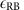) that closely approximates the gates’ average process infidelity. Because RB is relatively insensitive to unitary errors^27^ that dominate diamond norm error^28^ and have unpredictable consequences for FTQEC^20^, it cannot efficiently measure diamond norm error to high precision. This makes it nearly impossible to demonstrate suitability for FT using RB alone, unless errors are assumed to be strictly incoherent. There are variants of RB that characterize and report additional parameters, but none of them are well suited for diamond norm characterization or comparison to FT thresholds^29^,^30^,^31^.

We use a characterization method called gate set tomography (GST)^32^,^33^,^34^ to systematically debug and improve a 1-qubit trapped-Yb^+^-ion quantum information processor, and—finally—to demonstrate with very high confidence that all three of its quantum logic operations surpass a proven threshold for FTQEC. GST provides a full and extremely accurate tomographic description of every gate, complete with statistical confidence bounds. We use this information to iteratively improve our single-qubit operations and to place tight bounds on the diamond norm error of the final gates, producing the first single-qubit gates whose errors are demonstrably below a rigorous threshold for fault-tolerant error correction.

This is not a demonstration of FTQEC, which requires not just single-qubit gates, but also high-fidelity two-qubit gates, repeatable measurements and (of course) more qubits. However, the GST methods that we use here to demonstrate 1-qubit gate errors below the threshold do generalize to 2-qubit gates, to the characterization of repeatable measurements, and to important properties of multiqubit systems such as crosstalk. So, while pushing single-qubit gate errors below the threshold is only one step toward achieving FTQEC, it is an important one.

## Results

### Gate set tomography

Our goals are (1) to implement quantum operations satisfying a FTQEC threshold and (2) to ‘prove’—that is, demonstrate conclusively—that we have done so. Genuine proofs are the domain of mathematics. In experimental science, the highest achievable standard is to provide experimental data (or summary statistics) that (1) are consistent with the desired outcome; and (2) are inconsistent with any other plausible theory, and thus rule out all alternatives to some high level of confidence. Our intent is not to provide an exclusive protocol for such demonstrations, but rather to establish that GST is sufficient to do so.

While RB and quantum process tomography^35^,^36^,^37^ can be used in this fashion, they each face nontrivial obstacles. RB’s insensitivity to unitary errors makes it a poor tool for bounding worst-case error rates (diamond norms). In process tomography^5^,^38^, small calibration errors in the gates used to implement different measurements propagate to the final results, invalidating them. GST is a self-calibrating tomography protocol that solves both of these problems. GST protocols based on short quantum circuits were developed at IBM^38^ and Sandia^32^. The long-sequence GST protocol demonstrated here is orders of magnitude more precise.

**Figure 1:**
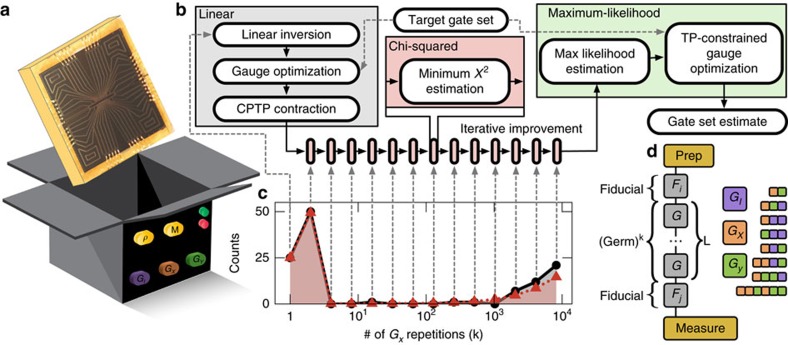
Gate set tomography is a robust procedure to characterize as-built qubits. (**a**) GST models the qubit (a single Yb^+^ ion localized in a linear surface electrode ion trap) as a ‘black box’ with a classical interface, and is agnostic to its physical details. (**b**) Flow chart of GST analysis. Its core is an iterative *χ*^2^ minimization, over data from increasingly long gate sequences, seeded with a linear inversion estimate. The final step, likelihood maximization, produces an accurate and unbiased estimate of the gate set. (**c**) A subset of the nearly 5,000 data points taken: observed ‘bright’ counts (black) for sequences of the form 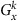 and the GST estimate’s prediction (red; see Fig. 3). Deviations from ideal gates appear only at *k* >1,000. (**d**) GST achieves high precision from periodic sequences based on short ‘germs’. Here the 11 germs used for this experiment are shown (coloured boxes), as is the ‘fiducial sandwich’ form of a general GST sequence.

GST relies on two assumptions: (a) the system being characterized is a qubit with a 2-dimensional Hilbert space; (b) each gate operation is stationary and Markovian. It treats the qubit as a black box with operation buttons (one for initialization, one for measurement and the rest for gate operations) as shown in Fig. 1a, and self-consistently determines all operations up to a choice of basis (a gauge; see ‘Methods’ section). It can also detect and quantify violations of these assumptions (see next subsection).

In GST, the real (noisy) gates are modelled as trace-preserving linear maps on density matrices (TP maps). Such maps must be completely positive to be physical, and thus are usually referred to as ‘CPTP maps’; for technical reasons, we do not always impose the CP constraint in GST, but otherwise these maps are functionally the same as CPTP maps.

The qubit’s quantum state *ρ* is a four-element vector 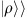 in the vector space of 2 × 2 Hermitian matrices (Hilbert–Schmidt space)^32^, and each gate is a 4 × 4 matrix *G* that acts on 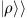 by left multiplication (that is, 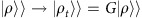). Measurement is represented by a two-outcome positive operator-valued measure (POVM) {*E*, −*E*}. Our target state preparation and measurement (SPAM) are 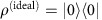 and 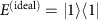.

Data for GST come from gate sequences (quantum circuits), each comprising: (1) initialization, (2) a series of gates and (3) measurement. Each sequence is repeated *N* times, and the frequency of 0/1 counts is recorded. In the experiments reported here, we implemented and used the set of gates {*G*_*I*_, *G*_*X*_, *G*_*Y*_}, but GST can analyse any gate set rich enough to prepare an informationally complete set of probe states and measurements.

GST analysis proceeds as shown in Fig. 1b. First, a specific set of short sequences is analysed by linear inversion (see ‘Methods’ section) to get a rough estimate of the gates and SPAM operations. This estimate has an unavoidable gauge freedom; every observable probability is invariant under


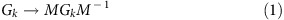



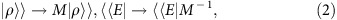


for any invertible matrix *M*. We choose a gauge that makes the estimated gates as similar to the target gates as possible (see ‘Methods’ section). If the rough estimate is not already completely positive, we truncate each gate to the nearest CP map, to ensure physically valid probabilities in the next step. Next, using the rough estimate as a starting point, we iteratively add more data. In the *m*th iteration, we add data from gate sequences of length 2^(*m*−1)^ into the pool, then numerically adjust the estimate to minimize the *χ*^2^ divergence between the observed frequencies and estimated probabilities. This ‘min-*χ*^2^’ estimate is then used as the seed for a numerical maximization of the likelihood function 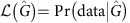. Finally, we perform another gauge optimization to maximize similarity to targets.

The GST gate sequences (see Fig. 1d) are chosen to (collectively) amplify every physical parameter in the gate set. Short sequences called germs are repeated many times, and these ‘germ power’ sequences are pre- and post-fixed by each of six fiducial sequences. In this work, we use six fiducial sequences, {∅, *G*_*X*_, *G*_*Y*_, *G*_*X*_*G*_*X*_*G*_*X*_, *G*_*Y*_*G*_*Y*_*G*_*Y*_, *G*_*X*_*G*_*X*_}, where ∅ denotes the null sequence, and *G*_*X*_ (*G*_*y*_) are noisy *π*/2 rotations about *x* (*y*). These fiducials map *ρ* and *E* to (approximately) the six Pauli eigenstates, defining an informationally complete experimental reference frame. For further details on sequence design and a complete list of all experiments performed, see ‘Methods’ section.

### Experiment

Our qubit is a single ^171^Yb^+^ ion in a state-of-the-art linear surface ion trap (Fig. 1a). Ions are trapped by photoionizing neutral ytterbium vapour that reaches the trapping volume through a slot from the back of the surface trap chip. The qubit is encoded in the hyperfine clock states of the ^2^*S*_1/2_ ground state of ^171^Yb^+^: 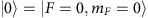, 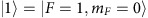. Standard laser cooling techniques are applied to Doppler cool the ion and prepare it in the |0> state^39^. Standard fluorescence state detection^39^ is used to measure in the 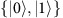 basis.

Three logic gates—*G*_*I*_ (the idle or identity gate), *G*_*X*_ (a *π*/2 *X* rotation) and *G*_*Y*_ (a *π*/2 *Y* rotation)—are realized by using a microwave horn to apply pulses near-resonant with the 12.6428 MHz separation of the qubit levels. Broadband composite pulses (BB1)^40^,^41^ are employed to minimize sensitivity to amplitude fluctuations in the microwave signal.

Using characterization procedures to debug and improve qubits has a long history. A Ramsey fringe or Rabi oscillation experiment is a kind of limited tomography, combined with physical intuition, which is used to improve the quality of the quantum logic operations. In typical tune-up procedures, different types of these experiments are iterated, until the qubit gates are deemed good enough to proceed.

Various improvements to this generic tuning-up scheme outlined above have been offered, including augmenting oscillation experiments to detect microwave pulse distortions^42^, designing small sequences for error amplification^43^, supplementing sequence experiments with RB to do detailed noise spectroscopy^44^ or replacing them with iterative RB to guide the system toward higher RB fidelity operations^45^. All of these techniques, and other experiments combined with physical intuition, can be and have been used to produce qubits with very high-fidelity operations. In comparison, GST has the distinct advantage in that it includes all experiments necessary for full and loophole-free qubit characterization. It can be though of as systematic statistical inference on a provably sufficient set of Rabi/Ramsey experiments.

**Figure 2:**
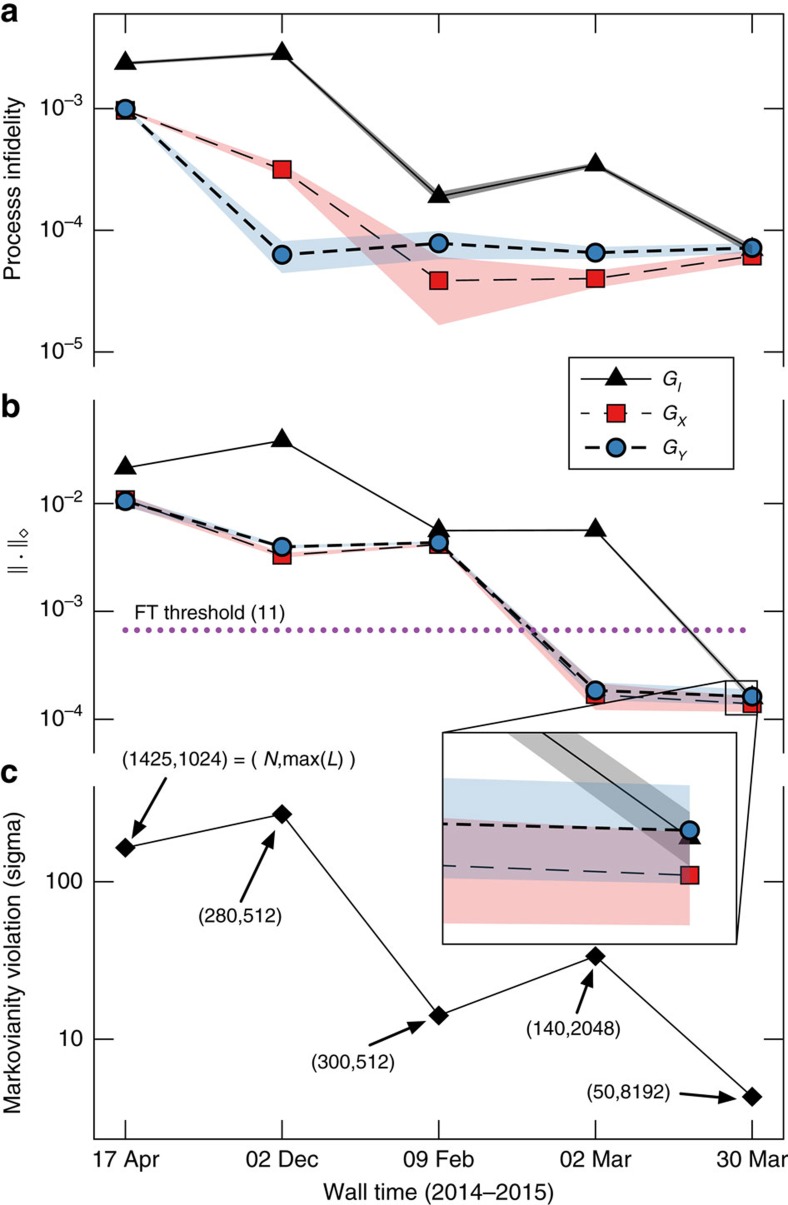
Progressive improvement of quantum operations. Over the course of ∼1 year, we used GST to improve our qubit, ending with clearly sub-threshold error rates. All metrics are computed using GST estimates based on data taken at the given time, but analysed using best available algorithms at publication time. (**a**) Process infidelities of the three gates versus wall time. (**b**) Diamond norm distance from estimated gates to targets versus wall time. Experiments from March 2015 surpass the best-known diamond norm threshold of 6.7 × 10^−4^ with 95% confidence, satisfying the threshold for fault tolerance established in ref. 22. (**c**) Violation of Markovian model (in s.d.'s *N*_*σ*_) versus wall time (see the section ‘Quantifying non-Markovianity’ for details). Non-Markovian noise was progressively eliminated (for example, by adding drift control and dynamical correction; see main text), guided by GST.

We used GST to analyse systematically and improve our trapped-ion qubit operations over the course of five experimental runs from 17 April 2014–30 March 2015. Experiments #1–2 used the Sandia Thunderbird trap^46^, and Experiments #3–5 used Sandia’s high-optical-access (HOA-2) trap. Figure 2a summarizes the gates’ steady improvement over this period by tracking their process infidelities^47^, which corresponds to the RB error rate^48^.

**Figure 3:**
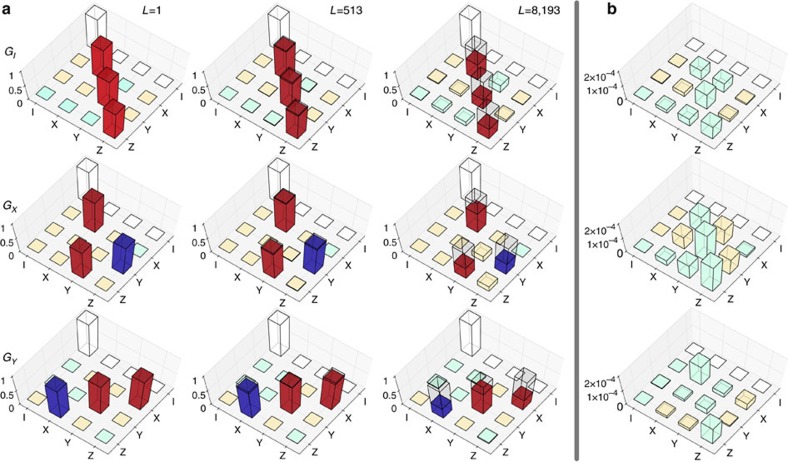
Process matrices and error generators for the final gates implemented 30 March 2015. (**a**) GST estimates of the *G*_*I*_, *G*_*x*_ and *G*_*y*_ gates, shown as superoperators in the basis of Pauli matrices, and based on data from gate sequences out to length 8192. For each estimate 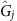, we also show 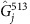 and 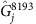 to emphasize errors. Bar height shows absolute value of matrix elements. White bars are fixed by the TP (trace-preserving) constraint, red ones should (ideally) be +1, blue ones should be −1 and beige/teal ones should equal 0 but are positive/negative, respectively. Wireframes indicate the ideal (target) gates for comparison. (**b**) Error generators for each gate, using the same colours as **a**. We define these as 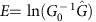, where 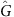 is the estimate and *G*_0_ is the target.

Experiment #1 detected severely non-Markovian behaviour. We sought to address this by stabilizing the microwave amplifier’s temperature, and stabilizing microwave *π*-times using active feedback (drift control), as described in ‘Methods’ section. Experiment #2 showed improved fidelity in the *G*_*X*_ and *G*_*Y*_ gates, but no reduction in non-Markovianity. We then moved our qubit to the HOA-2 trap, and improved trap stability. In Experiment #3, GST showed significant improvements in fidelity and Markovianity, and that *G*_*I*_ remained worse than the other gates. To improve it, we changed *G*_*I*_ from ‘do nothing for one clock cycle’ to the dynamical decoupling pulse sequence *X*_*π*_*W*_1.25*π*_(*X*)_−*π*_*W*_1.25*π*_, where *X*_*π*_ and *Y*_*π*_ denote *π* rotations around *X* and *Y*, respectively, and *W*_1.25*π*_ means ‘wait for the duration of a 1.25*π*-pulse’. We also applied active drift control of the qubit frequency, and improved the calibration of the BB1 pulse sequences. Experiment #4 showed reduction of coherent errors in *G*_*X*_ and *G*_*Y*_, but persistent non-Markovian errors in *G*_*I*_. After we upgraded *G*_*I*_ to the second-order dynamical decoupling sequence *X*_*π*_*Y*_*π*_*X*_*π*_*Y*_*π*_ (ref. 49), Experiment #5 demonstrated uniformly excellent gates. Subsequent analysis indicates that the improved performance of *G*_*I*_ stemmed largely from the constant duty cycle of the microwave system, rather than from the intrinsic properties of the decoupling sequence used. This emphasizes that GST can identify specific errors, but not necessarily their cause. The estimated process matrices for the gates are shown in Fig. 3a. Figure 3b shows the error generators, defined as 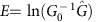, where 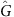 is the estimate and *G*_0_ is the target.

### Demonstrating suitability for FT

Useful quantum computation is expected to require fault-tolerant error correction. The most important milestone for a quantum operation is, therefore, ‘Is it suitable for use in FTQEC?’ Operations that induce too much error will cause FTQEC protocols to fail. Demonstrating conclusively that gates are suitable for FT requires: (1) establishing a sufficient condition for the gates to not induce failure; and (2) showing that the gates satisfy that condition, with high confidence, by means of experimental data that are inconsistent with all gates that do not satisfy the condition.

Demonstrating suitability for FT using infidelity alone is hard. Threshold theorems against general errors (arbitrary CP maps) are stated in terms of the diamond norm distance between the real and ideal gates^24^,^50^,


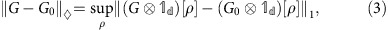


where *d* is the system’s Hilbert space dimension, 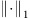 is the trace norm and the supremum is over density matrices *ρ* with dimension *d*^2^ (ref. 51). Because the diamond norm error can be as large as 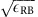 (refs 27, 28), even a spectacular RB result like 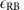
=10^−6^ (ref. 15) only establishes an upper bound of 10^−3^ on the diamond norm. The best-known proof of FT against general noise^20^ derived a threshold of 2.3 × 10^−5^ against stochastic noise, and generalized it to a diamond norm threshold of ∼10^−5^ against general (coherent) noise. This was subsequently improved to 1.94 × 10^−4^ (ref. 21) and finally to 6.7 × 10^−4^ (ref. 22), the highest (currently) proven threshold against general noise.

Unlike RB, GST enables direct computation of the diamond norm between the estimated and target gates (we use a semidefinite program^52^). Figure 2b shows the diamond norm error of our gates over time, culminating on 30 March 2015 in diamond norm error rates (with 95% confidence intervals) of (1.58±0.15) × 10^−4^, (1.39±0.22) × 10^−4^ and (1.62±0.27) × 10^−4^ for *G*_*I*_, *G*_*X*_ and *G*_*Y*_ respectively. All three gates surpass the threshold with 95% confidence. (In point of fact, they surpass even the older 1.94 × 10^−4^ threshold with 95% confidence.)

We note that, although we only demonstrated Clifford operations, and non-Clifford operations are needed for universal control, FTQEC is possible only using Cliffords. Furthermore, we can still extrapolate the performance of non-Clifford gates (for example, a T gate) in our system. A pessimistic estimate of the error on an *X π*/4 rotation, for example, would simply be the same as the error on the *X π*/2 gate that we characterized. This is because implementing the *X π*/4 gate in practice is equivalent to running the *X π*/2 gate for a shorter duration.

### Quantifying non-Markovianity

In real experimental systems, repeated quantum operations are never actually identical. For example, experimental imperfections in the stability of the system may cause quantum operations to drift over time. Collectively, we refer to all such non-repeatability as non-Markovianity. It represents a significant potential problem for FT, as proofs of FT thresholds are typically carried out using Markovian error models. So, to be confident that a gate set is suitable for FTQEC (that is, achieves a FT threshold), we would like to demonstrate that non-Markovian behaviour is absent. This is not feasible, for two reasons. First, all physical systems (including qubits) are at least a tiny bit non-Markovian. Second, ‘non-Markovian noise’ is so general that there is always some conceivable mechanism that would elude detection by any protocol (not just GST). Our goal is to reduce detectable non-Markovian behaviour to the point where its visible effects are consistent with the FT threshold.

We use GST results to debug non-Markovian effects and achieve this goal, as illustrated in Fig. 2c. Doing this is nontrivial, because neither GST nor process tomography is actually designed to characterize non-Markovianity. In GST’s underlying model, the qubit is Markovian: its state at time *t*+1 is determined completely by (1) its state at time *t* and (2) the operation applied at time *t*. This assumption is far reaching. It implies that noise in the logic gates is stationary, uncorrelated in time, memoryless and independent of context (for example, what gates were recently applied). It implies that the gate operations can (for a single qubit) be represented as static 4 × 4 superoperators, and that state preparation and measurements may each be represented as static four-dimensional vectors and dual vectors (respectively), in Hilbert–Schmidt space.

As indicated above, the Markovian assumption is not strictly true for any experimental system. In addition to slow drift, there may be correlations between errors in consecutive gates, and the ‘qubit’ may not even be a two-level system (for example, due to leakage levels). These are all examples of non-Markovianity, and lie outside the GST model.

In principle, all guarantees about GST are void in the presence of non-Markovian noise, as there are no process matrices to measure or report. However, for many typical non-Markovian behaviours, GST degrades in a quantifiable way. These kinds of non-Markovian noise cause data that are consistent with no Markovian gate set, and this failure to fit the data can be quantified. Since data generated by any Markovian model could be fit with predictable accuracy, significant badness-of-fit can be interpreted as violation of the model and therefore as non-Markovianity, though the particular type cannot be easily identified. As long as the data appear sufficiently Markovian, the GST estimate will be fairly reliable and have significant predictive power.

To quantify non-Markovianity, we consider the log-likelihood (equation (13))


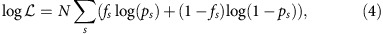


where *f*_*s*_=*n*_*s*_/*N*. The best conceivable fit to a data set would be one where *p*_*s*_=*f*_*s*_ for every sequence *s*. Thus, the entropy of a data set is an upper bound on log 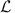,


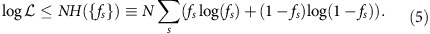


We define the quantity 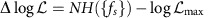.

Standard properties of maximum-likelihood estimation theory^53^ imply that if

1. The data were in fact generated by some gate set,
2. There are *N*_*p*_ free parameters in the gate set, and
3. The data set contains *N*_*s*_>*N*_*p*_ distinct gate sequences

then as *N*→∞, 2Δlog 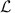 is a random variable with a *χ*_*k*_^2^ distribution, where *k*=*N*_*s*_−*N*_*p*_. This means that its expected value is 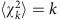, and its RMS variance is 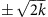. Thus, if the fit is ‘good’, then 2Δlog 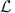 should lie roughly within the interval 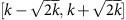. Hence, by comparing the difference 2Δlog 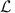
−*k* to 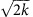, we can determine how well the Markovian model was able to fit the data.

We quantify goodness-of-fit by *N*_*σ*_, the number of s.d.’s from the expected mean the expected mean the log-likelihood score is:


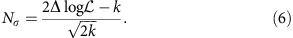


**Figure 4:**
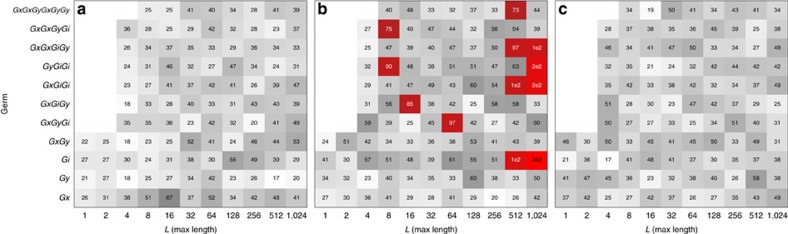
log 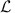 box plots for GST fits for three data sets. In each individual box, 2Δ log 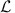 is summed over a set of 36 different gate strings. If the underlying system is Markovian, 2Δ log 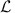 is (approximately) a 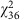 random variable. The box colour indicates the 2Δ log 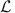 score for that collection of sequences. Grey indicates score values that are expected due to statistical fluctuations, while red indicates significant model violation at 95% confidence. That is, if the gates are Markovian, the probability of observing even one red square is at most 5%. Note that certain germs are too long to appear at *L*=1, 2, 4. (**a**) log 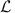 box plot for simulated Markovian data. (**b**) log 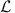 box plot for experimental data from 2 March 2015, with indications of strong non-Markovianity. (**c**) log 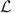 box plot experimental data from 30 March 2015, with vastly decreased non-Markovianity.

We can also calculate 2Δlog 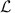 for individual experiments or subsets of gate sequences. Figure 4 illustrates this, where 2Δlog 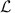 is shown for every individual experiment associated with each power of each germ (for a total 36 experiments per collection, due to six preparation fiducials and six measurement fiducials). This analysis makes it possible to see whether non-Markovianity increases with sequence length (it usually does, because longer sequences amplify slowly varying noise), and which sequences are particularly inconsistent with the best Markovian fit.

Figure 4 compares 2Δlog 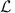 scores for a simulated (perfectly Markovian) data set to those for two experimental data sets, one from 2 March 2015, and the other from 30 March 2015. The March 2 experimental data set is highly non-Markovian, while the March 30 data set looks very similar to the simulated data set. These data demonstrate the degree to which we are able to stabilize our qubit and reduce non-Markovian effects. In the final run on March 30, Markovianity is violated only at the 4*σ* level. While this is statistically significant—it implies with high confidence that the gates are not perfectly Markovian—it is not practically significant. To see this, recall that this is an extraordinary sensitive experiment, as witnessed by the fact that the error bars on the diamond norm are ±2 × 10^−5^. This sensitivity extends to non-Markovian behaviour as well. Reducing the sensitivity of the experiment by a factor of 4 (either by reducing maximum *L* by a factor of 4, or by reducing *N* by a factor of 16) would render the non-Markovianity undetectable, at the cost of increasing the error bars by a factor of 4 to ±8 × 10^−5^. This implies that the observed non-Markovianity is effectively equivalent to less than 10^−4^ additional diamond norm error, which is comfortably below the threshold.

### Comparison to randomized benchmarking

As of this writing, RB is the de facto standard in qubit characterization. As a consistency check, we perform RB simultaneously with the final GST experiment (by interleaving the GST and RB sequences over the entire period of experimentation), to see whether GST correctly predicted the results of RB. We follow the experimental and analysis procedure of ref. 54, and use RB sequences ranging in length from two gates to 1,970 gates.

Strictly speaking, RB measures the error rate per Clifford operation. Our Clifford operations are, as is usual, compiled into elementary {*G*_*i*_, *G*_*x*_, *G*_*y*_} gates, with an average of 3.125 elementary gates per Clifford. Analysis of the data in strict accordance with the literature (that is, plotting survival probability versus # of Cliffords in the sequence) yielded an experimental error rate of (1.65±0.03) × 10^−4^ per Clifford operation. Dividing this by 3.125 (a questionable but common practice) suggests a per-gate error rate of about 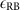
=(5.28±0.10) × 10^−5^.

**Figure 5:**
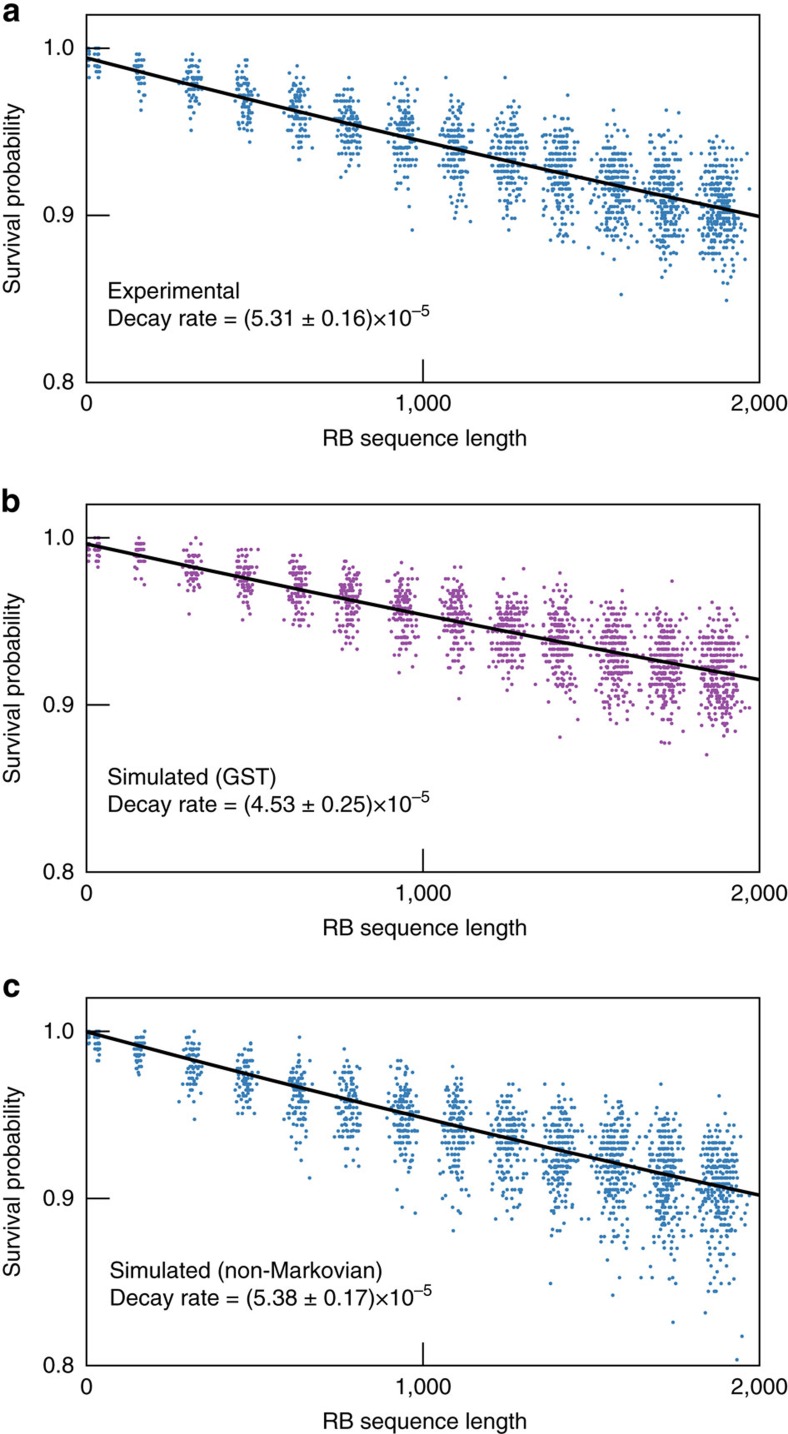
Randomized benchmarking results. Coloured dots are experimental or simulated data points; lines are exponential decay fits to the data. (**a**) Experimental RB data. (**b**) RB data simulated using the gate set 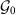 derived from experimental GST results. (**c**) RB data simulated using the non-Markovian gate set 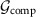. Here 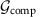 is generated using the composite Nyquist-limited noisy gate set model proposed in the section ‘Comparison to Randomized Benchmarking.’ This model toggles between slightly over- and under-rotated gates with every gate application, which is a reasonable scenario for our qubit due to our use of DCG. The experimental RB decay rate is (5.31±0.16) × 10^−5^, which is indistinguishable from 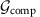
’s RB decay rate of (5.38±0.17) × 10^−5^, but distinct from 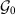
’s RB decay rate of (4.53±0.25) × 10^−5^, demonstrating the plausibility of our non-Markovian model in explaining the apparent discrepancy between experimental RB and GST.

However, our main goal is to compare the RB data with GST’s predictions for it. For this purpose, we find it more informative to fit (and plot; see Fig. 5) the observed probabilities versus the number of elementary gates in the sequence. All the rest of the analysis in this section is based on this analysis method, which yields a per-elementary gate RB error rate of 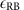
=(5.31±0.16) × 10^−5^. Error bars are 95% confidence intervals. The experimental error bars are calculated via non-parametric bootstrap (by resampling the experimental data with replacement). We then simulate those RB experiments using the GST estimates. The GST results predict an RB error rate of 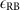
=(4.53±0.25) × 10^−5^ (see Fig. 5a,b). The simulated error bars are calculated via parametric bootstrap. (The GST estimate is used to generate many sets of simulated GST experiments, each of which in turn yields a new GST gate set estimate. This ensemble of estimates then generates an ensemble of simulated RB decay rates, from which the simulated error bars are derived).

While these decay rates are nearly identical, there is a statistically significant discrepancy. The most obvious explanation is a flaw in the GST analysis, but we find that extensive simulations with known Markovian gates rule this possibility out. We believe that the discrepancy stems from physical causes—that is, from non-Markovian noise. The most common form of non-Markovian errors is low-frequency drift, which manifests in both RB and GST as coherent errors that remain nearly fixed over the course of any one sequence, but change from sequence to sequence (and between repetitions of a single sequence). In the presence of such effects, GST typically overestimates the RB decay rate, because GST amplifies coherent errors to which RB sequences are relatively insensitive (Markovian or not). Thus, GST typically reports a higher rate of Markovian noise in a quixotic attempt to fit its data, while RB simply does not see the noise.

But in this experiment, we observe the opposite effect. Instead of over estimating the RB error rate, GST under estimates it. While the exact cause remains uncertain, we observe that this behaviour is completely consistent with anti-correlated noise (each gate flips between under- and over-rotation at each application) induced by dynamically corrected gates (DCG)^49^.

Here is a concrete model that reproduces this behaviour: Consider a unitary error that varies in time—but instead of varying slowly, it oscillates at the system’s Nyquist frequency (that is, flips sign every clock cycle). For simple gates implemented with a single pulse, this would be highly implausible. In this experiment, however, we implement DCG. The simplest DCG is a dynamically corrected idle gate (our *G*_*I*_). This is nothing but dynamical decoupling—periodic *X*_*π*_ pulses that echo away small *Z* rotations. Such sequences create a ‘toggling frame’ for the qubit that flips sign twice per clock cycle. Any timing or amplitude errors in the pulses can leave a residual error that flips sign every clock cycle, making a plausible noise model for a DCG.

We model this effect by augmenting the qubit state space with a classical binary variable *q*∈{−1, +1}. We define a composite gate set 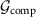, based on a standard gate set 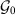, which consists of two single-qubit gate sets 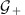 and 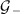 that act conditionally on the value of the classical bit *q*, which flips every time a gate is applied. These gate sets are identical to 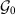, except that the *G*_*X*_ and *G*_*Y*_ elements of 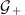 have a fixed, slight over rotation by an angle *θ*, while the *G*_*X*_ and *G*_*Y*_ elements of 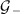 have a fixed, slight under rotation by *θ*. At the beginning of each simulated experiment, *q* is chosen randomly.


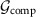
 acts on an eight-dimensional state space, and data generated from it is not fully consistent with any Markovian single-qubit gate set. But GST can be applied to that data, and will find the Markovian single-qubit gate set that fits it best. (Indeed, as no experimental system is perfectly Markovian, this is in essence what GST always does).

We generate simulated data, with finite-sample error, for all the GST and RB experiments performed on 30 March 2015. For this simulation, 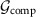 is defined by setting 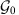 equal to the GST estimate from 30 March 2015, and setting *θ*=1.25 × 10^−2^.

Analysing the GST data generated by 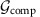 yields an estimated gate set almost identical to that obtained from experimental data 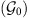. All but two of the 36 free gate matrix elements are within the 95% confidence intervals assigned to 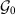, and the three remaining elements are at most 0.05*σ* outside them. Every gate is within 4.4 × 10^−5^ (in diamond norm) from the corresponding gate in 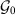. We conclude that GST, as performed, cannot distinguish the composite model 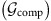 from 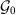.

The RB data simulated with 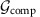 also matches the experimental RB data almost perfectly, yielding an RB error rate of (5.38±0.17) × 10^−5^ that is statistically indistinguishable from the experimentally observed RB decay rate of (5.31±0.16) × 10^−5^. Both data sets (experimental and simulated-by-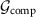) are shown in Fig. 5, along with RB data simulated from 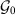. We conclude that RB observes significantly different error rates for 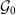 and 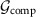.

This does not imply that our qubit really is described by 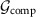, but it demonstrate a plausible non-Markovian model that is fully consistent with our data. There might be many other (different) non-Markovian models equally consistent with it. And while certain kinds of non-Markovian noise can be detected by RB^55^ and GST, neither GST nor RB are designed to function reliably in the presence of any non-Markovian noise, so neither of them is explicitly ‘right’ or ‘wrong’ for this case.

### The relative power of RB and GST

RB and GST share the common framework of data from gate sequences (circuits) that are (1) diverse, (2) repeated and (3) long. But they are distinguished by the kind of sequences performed. RB sequences are random, for the specific purpose of ‘twirling’ the noise. GST sequences are structured and periodic, for the specific purpose of amplifying errors.

This difference is fundamental. It makes RB intrinsically insensitive to coherent errors, which dominate the diamond norm error metric^27^,^28^. For example, suppose that one logic gate over-rotates by a small angle *θ*, while the others are perfect. In random sequences containing *L* applications of this gate, it will (by construction) be interleaved with other gates chosen randomly. The rotations by *θ* will add up incoherently, producing (on average) a total rotation of 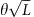, and therefore an error probability of *Lθ*^2^. Thus, a coherent error by *θ* appears (in RB) as an incoherent error of probability *θ*^2^.

But circuits of practical interest are not random. Since not all ‘useful’ circuits are known at this time, it is wise to consider how errors affect arbitrary circuits in the worst (most fragile) case. For the example given above, the worst case is a periodic sequence in which the imperfect gate appears *L* consecutive times. Rotations add up coherently, the final angle is *Lθ* and the final error probability is *L*^2^*θ*^2^. So, for example, a 10^−3^ rotation can cause a 1% failure rate after just *L*=100 gates. In randomized circuits, the same failure rate would require *L*=10^4^ gates.

The diamond norm metric is a strict upper bound on the rate at which failure probabilities can grow, and so it takes account (by construction) of the worst-case behaviour given above. The diamond norm error for a small coherent error by angle *θ* is *O*(*θ*). Process infidelity (closely related to the RB error rate) does not account for worst-case behaviour, and the process infidelity for a small coherent error by angle *θ* is *O*(*θ*^2^).

GST intentionally implements a wide variety of periodic sequences, to ensure that at least one of them is approximately ‘worst case’ for every possible coherent error. This allows GST to detect coherent errors of size *θ* using sequences of length *L*=*O*(1/*θ*), repeated *O*(1) times. Detecting the same error with randomized sequences requires much long sequences of length *L*=*O*(1/*θ*^2^), or else a much higher number of repetitions (both of which correspond to orders of magnitude more time and effort).

Periodic sequences might be incorporated into RB, to make it more sensitive. Doing so, however, would eliminate its characteristic feature. Such a protocol would no longer be RB. On the other hand, there are several interesting variations of RB that retain its randomized nature, most notably interleaved benchmarking^29^, RB tomography^30^ and unitarity benchmarking^31^. While interesting in their own right, they are all subject to the same trouble: random gate sequences are much less sensitive to coherent errors than periodic ones, and therefore every form of RB is necessarily inefficient at detecting coherent errors.

Unitarity benchmarking is particularly interesting, since (unlike other forms of RB) it can separate coherent and incoherent errors, and therefore provide good information about diamond norm error rates. Unfortunately, it is (compared with GST) extremely inefficient at doing so.

Wallman *et al*.^31^ defined a quantity *u* (unitarity), which measures the rate of purity decay. They gave an RB-like protocol for measuring it, and pointed out that *u* and *r* together could be used to bound the diamond norm error. If


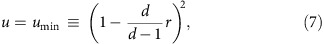


then the noise is purely incoherent, and the diamond norm error is *O*(*r*). If *u*−*u*_min_ is sufficiently small, then the errors are primarily incoherent, and the diamond norm error remains *O*(*r*).

However, the actual bounds (see ref. 56) are of the form


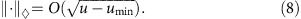


We have demonstrated that our gates’ diamond norm error is *O*(*r*) using GST. Doing the same thing using unitarity requires showing that *u*−*u*_min_=*O*(*r*^2^). But 1−*u* is itself an RB-type quantity, meaning that it appears as an error rate (in experiments that measure purity), and is measured using RB. As a result, showing that *u*−*u*_min_=*O*(*r*^2^) is equivalent to:

1. Performing standard RB to measure *r*, the decay rate of sequence fidelity.
2. Performing a different RB-like experiment to measure *r*′=1−*u*.
3. Demonstrating (based on those experiments) that *r*−*r*′=*O*(*r* ^2^).

For *r*=10^−4^ (the regime we access experimentally), this requires measuring both *r* and *r*′ to 10^−8^ precision. This is extraordinarily hard. The most efficient way to do it is using sequences of length *L*≈10^4^. These would yield survival probabilities around *p*_*L*_≈1/*e*. Achieving the necessary precision would require estimating *p*_*L*_ to ±10^−4^, which would require approximately *N*=10^8^ repetitions (because the uncertainty is 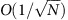). This is at least 10^6^ times more repetitions than would be required for standard RB, or for GST, and is completely impractical.

### Validating 10^−5^ accuracy with simulations

**Figure 6:**
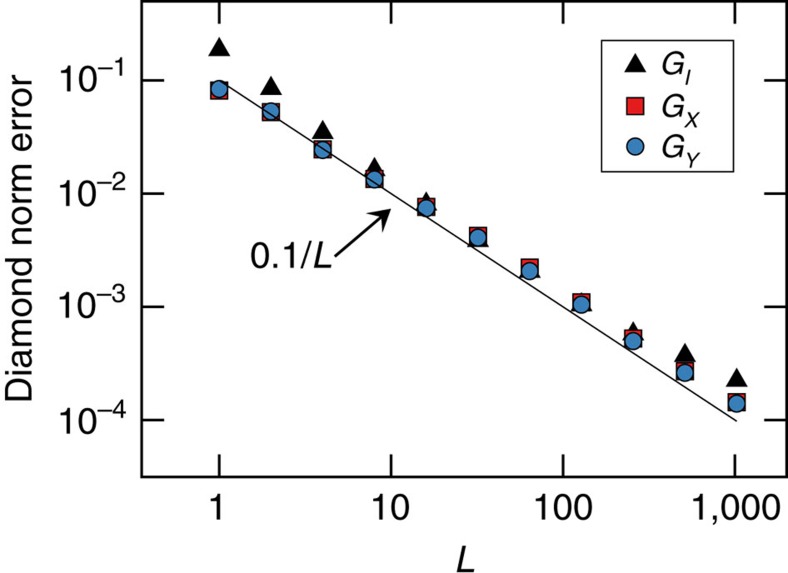
Confirmation of error scaling in GST. Here we show diamond norm difference between true and estimated gates in simulated GST with small unitary errors. Mean diamond norms are shown, averaged over 100 trials. Estimation error scales as 1/*L*, where *L* is the maximum sequence length in the data. Each trial uses *N*=50 samples per experiment.

We have claimed uncertainties (error bars/confidence regions—see ‘Methods’ section) of about 10^−5^ for diamond norms and process matrix elements. This is remarkable, and demands supporting evidence. To confirm this behaviour, we simulate GST experiments using (known) gate sets with unitary errors. The results (Fig. 6) confirm Heisenberg scaling: diamond norm distance between estimated and true gates decreases with the maximum sequence length (*L*) as 1/*L*. This scaling holds up to *L*≈1/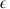, where 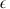 is the stochastic error rate. This is consistent with the ±∼10^−5^ observed error bars on diamond norm errors in our final experiment, for which *L*=8,192.

## Discussion

GST allows us to achieve high-quality gates in a trapped-Yb^+^-ion qubit, and to characterize it to unprecedented precision. Although lower-RB error rates have been reported in trapped-ion qubits^15^,^16^, our gates are the first to demonstrably surpass a rigorous FT threshold against general noise. GST is the first protocol that can efficiently demonstrate this important milestone and provide reliable feedback to debug and improve those gates.

Low-error single-qubit gates are just one of several critical achievements required to enable fault-tolerant quantum computing. Thus, this is only a first step. But GST—which can be generalized to 2-qubit gates and measurements—does answer one key and pressing question: ‘Once suitable operations have been achieved, how can their performance be verified for a critical, objective observer?’ RB can provide reliable information about process fidelity (which unambiguously captures stochastic or incoherent errors), but as of this writing, process fidelity is not known to be the relevant metric for FT. An exciting recent development in this area is the introduction of randomized compiling^57^, which has the potential to provably reduce the importance of coherent errors. But until and unless such techniques lead to a FT proof that is insensitive to them, and are confirmed to be practical in the context of FTQEC, coherent errors remain a point of concern. GST provides an efficient way to diagnose and bound all Markovian errors in gates.

## Methods

### Experimental details

In Sandia’s Thunderbird trap, ions were trapped 80 μm above the trap surface. Typical trap frequencies were 0.5, 1.8 and 2.3 MHz, for the axial and two radial modes, respectively. In the HOA-2 trap, ions were trapped 68 μm above the trap surface and trap frequencies of 0.5, 2.2 and 2.8 MHz were achieved. Typical trapping times were several hours for the Thunderbird trap and up to 100 h for the HOA-2 trap. Coherence times were measured to be ∼1 s in both traps, and were most likely clock-limited.

The microwave radiation used for qubit manipulation was generated by single-side band modulating the output of a 12.600 GHz dielectric resonator oscillator with the output of a direct digital synthesizer (DDS) near 42.812 MHz. The master clock for the dielectric resonator oscillator and DDS was generated by a rubidium frequency standard. The output of the single-side band modulator was amplified and directed parallel to the trap surface using a microwave horn. The microwave frequency and phase was controlled via the DDS and approximately square pulses were generated by switching the output of the DDS using a high-isolation rf switch. An offset was added to the constituent pulses of the BB1 pulse sequence to compensate for switching imperfections.

Drift control of the microwave π-time was realized by interleaving experiments in which the ion was initialized, exposed to a 10.5π microwave pulse, and measured. The π-time was adjusted after state detection; upon seeing 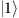, π-time was decreased by 0.625 ns, while upon seeing 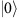, π-time was increased by 0.625 ns. For the next experiment, the π-time was then truncated to the time resolution of the experimental control (5 ns). Similarly, drift control of the qubit frequency was implemented by interleaving a Ramsey experiment in which the ion, after state initialization, is subject to: (1) a *G*_*X*_ gate, (2) a 25 ms wait time and (3) a *G*_*Y*_ gate. Upon state detection, the qubit frequency was adjusted by +8 mHz for a 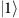 result, and by −8 mHz for a 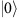 result.

### Linear GST

Linear-inversion GST (LGST) is a highly reliable but low-accuracy way to obtain an initial estimate of the gate set that serves as a seed for further refinement by long-sequence GST (see next subsection). LGST is essentially simultaneous ‘uncalibrated’ process and state tomography. By performing process tomography-like experiments on a set of gates, as well as the null operation (that is, the ‘do nothing for no time’ operation), LGST can provide rough estimates of all the gates involved, as well as the state preparation and measurement operations. LGST requires minimal assumptions about the various operations (unlike standard tomography), and computes its estimates using only basic linear algebra (the most complicated step is matrix inversion). A detailed explanation of the LGST procedure is provided in ref. 32; LGST is also described in ref. 33.

### Analysing long sequences in GST

GST incorporates data from long sequences in two stages. The first stage consists of several iterations, each of which performs a minimum-*χ*^2^ estimation. Each iteration takes the result of the previous iteration as a seed, and includes successively more of the long-sequence data. The second stage is a maximum-likelihood estimation, which is seeded from the first stage and uses all of the data. This procedure consistently avoids local minima in the objective function. In this section, we give the details of this algorithm (outlined in Fig. 1).

The iterative fitting procedure starts by fitting only data from the shortest gate sequences (which are easy to fit and insensitive to most non-Markovian noise), then successively adds longer and longer sequences (with base sequence length *L*≤1, 2, 4, 8, …). Since we get an estimate at each intermediate *L*, it is possible to quantify not just the goodness of the best fit, but how the goodness-of-fit behaves as longer and longer sequences are added in, which is useful for debugging.

At each step in the iterative process, we vary the gate set to minimize Pearson’s *χ*^2^ test statistic, which measures the discrepancy between a predicted probability (*p*) and an observed frequency (*f*). It is defined as


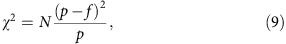


where *N* is the number of samples taken. In this analysis, *χ*^2^ is used to compare the set of probabilities predicted by a gate set (*p*_*s*_) and the frequencies obtained from a data set (*f*_*s*_). Each experiment (that is, gate sequence) *s* is associated to two probabilities: ‘plus’ has probability *p*_*s*_ and ‘minus’ has probability 1−*p*_*s*_. The *χ*^2^ of a single gate string *s* is


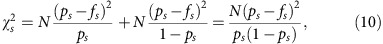


where *N* is the number of times the experiment *s* was performed, *p*_*s*_ is the probability of a ‘plus’ outcome as predicted by the gate set and *f*_*s*_ is the observed frequency of ‘plus’. The total *χ*^2^ for a data set 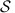 is just the sum


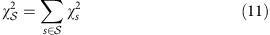


To estimate our gate set parameters, we minimize 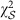 at each iteration using the Levenberg–Marquardt algorithim implemented in SciPy^58^.

The final stage in long-sequence GST analysis is a maximum-likelihood estimation (MLE), based on numerical optimization of the log-likelihood function log 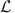. The log-likelihood for an *n*-outcome system with predicted probabilities *p*_*i*_ and observed frequencies *f*_*i*_ (*i*=1 … *n*) is given by:


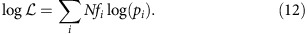


where *N* is the total number of counts. Like the *χ*^2^ statistic, log 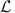 is used to compare the set of probabilities predicted by a gate set (*p*_*s*_) to the frequencies obtained from a data set (*f*_*s*_). Each experiment (that is, gate sequence) *s* is associated to two probabilities: ‘plus’ has probability *p*_*s*_ and ‘minus’ has probability 1−*p*_*s*_. The log 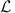 contribution of a single gate string *s* is


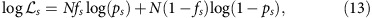


where *N* is the number of times the experiment *s* was performed, *p*_*s*_ is the probability of a ‘plus’ outcome as predicted by the gate set and *f*_*s*_ is the observed frequency of ‘plus’. The total log-likelihood for an entire data set is just the sum


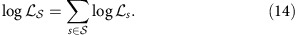


We find the maximum of this quantity using the same Levenberg–Marquardt algorithm as above, in order to compute the final (modulo gauge optimization) estimate of the gates.

LGST would be a perfect estimator in the absence of finite-sample error. However, it is inefficient with respect to accuracy. Like process tomography, its inaccuracy scales as 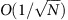, which means that achieving 10^−5^ error bars on all parameters would require around *N*=10^10^ repetitions of each experiment. Long sequences amplify errors proportional to *L*, enabling inaccuracy of 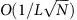 for all parameters. (This scaling breaks down for *L*≥1/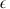, where 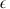 is the rate of stochastic decoherence. In our experiments, 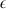
≤10^−4^, and we perform experiments as long as *L*=8192≈10^4^).

We use a hybrid algorithm (involving both min-*χ*^2^ and MLE) because each of its components have certain weaknesses. Empirically, we find that MLE is statistically well-motivated and avoids any bias, whereas *χ*^2^ optimization is numerically more stable and faster computationally but yields biased estimators, especially for the SPAM parameters. Our hybrid method combines both virtues, by using the more efficient and reliable min-*χ*^2^ algorithm to get a very good seed for the final (unbiased) MLE.

On a modern laptop, single-qubit GST with maximum *L*=1,024 can run in under 1 min; the analysis for maximum *L*=8,192 takes about 40 min.

### Selecting gate sequences for GST

The data that GST use to reconstruct a gate set come from performing gate sequences (that is, quantum circuits). Every gate sequence necessarily comprises (i) initialization, (ii) some gates and (iii) measurement (which yields a count that is recorded in the data set). The sequences used for GST have an additional structure:

1. Each GST sequence begins with a preparation fiducial sequence, and ends with a measurement fiducial sequence, with an ‘operation of interest’ sandwiched in the middle.
2. The ‘operation of interest’, which could in principle be any gate sequence, is chosen to be a germ power sequence—that is, a short ‘germ’ sequence, repeated an integer number of times.

Thus, every GST sequence is of the form 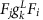, where *F*_*i*_ and *F*_*j*_ are preparation and measurement fiducials (respectively), *g*_*k*_ is a germ and *L* is an integer. *F*_*i*_ and *F*_*j*_ range exhaustively over a set of six fiducial sequences, while *g*_*k*_ ranges exhaustively over a set of 11 germs. In this section, we explain how the fiducials and germs are chosen.

The purpose of the fiducials is to prepare a sufficiently diverse set of input states and measurements to completely probe the operation of interest. This is achieved if (and only if) the input states 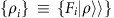 and the measurement effects 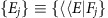 are both informationally complete (IC). A set of matrices is IC if and only if it spans the vector space 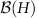 of matrices. This requires at least *d*^2^ linearly independent elements.

In general, any randomly chosen set of *d*^2^ states or effects will be IC. So, for single-qubit GST, we could choose *d*^2^=4 random fiducial sequences. However, while the resulting {*ρ*_*i*_} and {*E*_*j*_} will almost certainly be linearly independent, they may be close to linearly dependent. This property is quantified by the spectrum of the Gram matrix 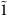, defined by


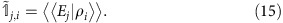


If either set fails to be IC, the Gram matrix will fail to have *d*^2^ non-zero (to machine precision) singular values. As any one of the *d*^2^ largest singular values becomes small, inverting the Gram matrix on its support (as is required for LGST) becomes ill-conditioned, and finite-sample fluctuations in GST get amplified, causing poor accuracy.

We would like both preparation and measurement fiducials to be uniformly IC, meaning that they span 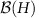 as uniformly as possible, and the smallest singular value of the Gram matrix is as large as possible. There exists a single-qubit uniformly IC set with only four elements (the SIC-POVM), but it cannot be generated with Clifford operations and stabilizer states. The smallest convenient uniformly IC set is the six-element set of stabilizer states (the eigenstates of *X*, *Y* and *Z*). We choose six fiducial sequences so that, if the gates are ideal, they will prepare the stabilizer states exactly. They are


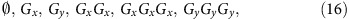


where ∅ indicates the null sequence (no gates).

Slightly imperfect gates will prepare states (and effects) that are close to the stabilizer states—and therefore close to uniformly IC, and almost as effective in probing the operation of interest. If the gates are sufficiently far from the targets, it can be detected by computing the singular values of the empirical Gram matrix, and then new fiducials can be chosen.

Once the fiducials are defined, we need to define ‘operations of interest’ for them to probe. By sandwiching any such operation between an exhaustive set of 36 fiducial pairs, we are essentially doing process tomography on the operation (although the algorithm for incorporating these data into the GST fit is more complex than simple process tomography).

The obvious operations of interest are the gates themselves (*G*_*x*_, *G*_*y*_ and *G*_*i*_). By probing each gate tomographically, and repeating each sequence *N* times, GST can estimate the gates to with 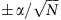 accuracy (for some constant *α*). To achieve higher accuracy, we do tomography on powers of the gates, by designating, for example, 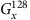 or 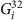 as an operation of interest. (Powers of 2 are chosen merely for convenience; any logarithmically spaced sequence of integer powers would work).

Repeating a gate *L* times—that is, performing sequences of the form 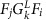
—amplifies errors in the gate itself. So, for example, if *G*_*x*_ is actually a rotation by *θ*=*π*/2+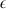, then 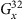 is a rotation by 32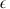. GST can now characterize that rotation to within 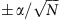, which equates to estimating *θ* to within 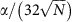. Raising gates to the *L*th power amplifies deviations by *L*, which in turn reduces estimation error by a factor of *L*.

However, simple repetition of *G*_*x*_ does not amplify every error. For example, suppose that *G*_*x*_ is in fact a *π*/2 rotation, but around the wrong axis, corresponding to the unitary map


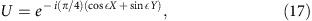


as opposed to the target unitary *e*^−*i*(*π*/4)*X*^. (*X* and *Y* indicate the Pauli operators *σ*_*x*_ and *σ*_*y*_).

This is a tilt error, and it is not amplified by 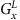. It’s easy to see this by observing that 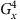
=1, so the error cancels itself out after just four repetitions.

More sophisticated sequences are needed to amplify tilt errors. For this example, it is sufficient to probe *G*_*x*_*G*_*y*_. Assuming (for now) that *G*_*y*_ is a perfect *π*/2 rotation around *y*, *G*_*x*_*G*_*y*_ is a rotation by 2*π*/3+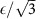. Therefore, performing (*G*_*x*_*G*_*y*_)^*L*^ amplifies the deviation 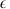 by a factor of *L*, and setting it as an operation of interest allows GST to estimate 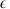 to within 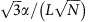. The short sequence *G*_*x*_*G*_*y*_ is a germ, and repeating it *L* times yields a germ power sequence that can be sandwiched between fiducials to equip GST with high sensitivity to the parameter 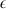.

The general situation gets rapidly complicated—for example, if *G*_*y*_ is not perfect, then *G*_*x*_*G*_*y*_ alone cannot distinguish between *Y* tilt in *G*_*x*_ and *X* tilt in *G*_*y*_. Each germ is sensitive to some nontrivial linear combination of gate set parameters. To choose a set of germs, we list the possible germs (that is, all reasonably short sequences), and for each germ *g* we identify what linear combination of parameters it amplifies. We do this by computing a Jacobian,


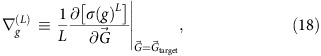


where *σ*(*g*) is the gate sequence product for germ *g* (obtained by just multiplying together the process matrices), and 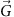 is a vector containing all the parameters of the gate set (for example, the elements of all the process matrices).

In the single-qubit case, *σ*(*g*) is a 4 × 4 matrix, and 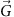 is 48-dimensional because it contains the elements of three 4 × 4 gate matrices. Constraining all gates to be trace-preserving reduces the number of free parameters to 12 and 36 (respectively), so ∇_*g*_ is a 12 × 36 matrix. Its 12 right singular vectors indicate linear combinations of gate set parameters that *σ*(*g*) amplifies (when repeated *L* times), and the corresponding singular values quantify how much they are amplified. A zero singular value indicates a parameter that is not amplified at all (like the tilt error discussed above). A set of germs {*g*_1_ … *g*_*N*_} is, collectively, described by a Jacobian


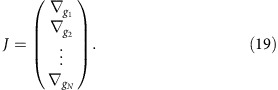


Our goal is to choose germs that provide high sensitivity at ‘large’ values of *L*. In practice, it is not useful to make *L* larger than 1/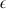, where 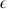 is the rate of stochastic or depolarizing noise. To select germs, however, we ignore this effect and make the simplifying assumption that the gates (and therefore *σ*(*g*)) are reversible (a good approximation when 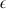 is small). Under this assumption, it is possible to define the *L*→∞ limit of the Jacobian in equation (18). Using the product rule, and assuming that all the gates are unitary (and therefore *σ*(*g*)^−1^=*σ*(*g*)^†^),


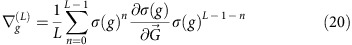



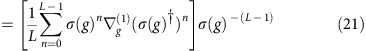


As *L*→∞, the average over all powers *n* of *σ*(*g*) twirls 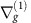. By Schur’s lemma, the effect of twirling is to project 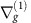 onto the commutant of *σ*(*g*)—that is, onto the subspace of matrices that commute with *σ*(*g*). Furthermore, multiplication by the unitary *σ*(*g*)^−(*L*−1)^ is merely a change of basis, and has no effect on the right singular vectors or the singular values of ∇_*g*_. So, up to an irrelevant change of basis:


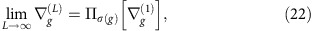


where Π_*σ*(*g*)_ is the projection onto the commutant of *σ*(*g*).

This framework defines a notion of informational completeness for germs. A set of germs {*g*_*i*_} is amplificationally complete (AC) if and only if the right singular rank of its Jacobian equals the total number of physically accessible (gauge-invariant) parameters in the gate set. For a general set of three single-qubit trace-preserving gates, a gauge transformation is *G*_*k*_→*TG*_*k*_*T*^−1^ where *T* is an invertible trace-preserving superoperator, so there are 12 gauge parameters and 36−12=24 gauge-invariant parameters. To build an AC set of germs, it is sufficient to add germs to the set until its Jacobian has rank 24. By constructing a complete set of infinitesimal gauge transformations, we can actually construct the projector Π_*g*−*i*_ onto the (local) space of gauge-invariant perturbations to the gate set 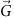.

We then optimize this set numerically, by adding and removing germs (taken from an exhaustive list of all short sequences), and only keeping a modification if it lowers a certain score function. (For single-qubit GST, we find it convenient to search over all germs of length ≤6. However, this set of candidates need not be exhaustive. (A larger gate set, for example, would generate a prohibitively large exhaustive candidate set). We have used randomly chosen subsets as candidate sets and gotten similar results). The score function is


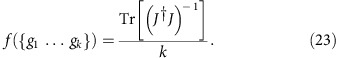


**Figure 7:**
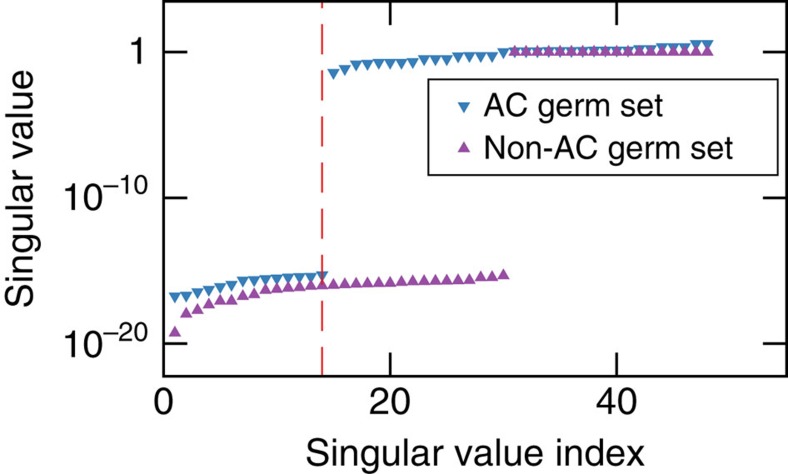
Sensitivity analysis for germ selection. Here we show the sorted singular values of the Jacobian matrix for different germ sets for the standard {*G*_*X*_, *G*_*Y*_, *G*_*I*_} gate set. Each singular value of the Jacobian corresponds to a gate set parameter; a large singular value indicates that the chosen germ set provides GST with sensitivity to that parameter. Given that there are always experimentally inaccessible gauge parameters, it is impossible to be sensitive to all parameters. The dashed red line indicates the number of gauge parameters for this gate set (14). The blue triangles are singular values for a Jacobian with the amplificationally complete 11-germ set used for the March 2015 GST runs; all singular values corresponding to non-gauge parameters are large. The purple triangles are singular values for the three-germ set containing just the bare gates *G*_*x*_, *G*_*y*_ and *G*_*i*_. It is amplificationally incomplete, as indicated by the presence of near-zero singular values that correspond to non-gauge parameters.

This score estimates the mean squared error of estimation if a fixed number of counts are spread over the *k* distinct germs. Running this algorithm until it cannot improve the germ set any further produces the following set of 11 germs used in the final (March 2015) GST runs (see Fig. 7):


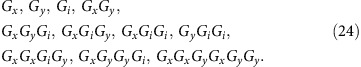


### The GST gauge, and how to set it

A gate set comprises: an initial density matrix *ρ* (represented as a Hilbert–Schmidt vector), a measurement effect *E* (represented as a Hilbert–Schmidt dual vector) and one or more gates *G*_*i*_ (represented as superoperators). But not every parameter in this representation is physically observable. A gate set has intrinsic ‘gauge’ degrees of freedom, because two distinct gate sets (or an entire manifold of them) can yield identical probabilities for all possible experiments. Gauge transformations alter a gate set’s elements without changing any observable probability. They take the form


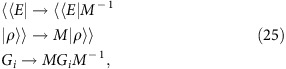


where *M* is any invertible superoperator. If (as usual) we consider only trace-preserving (TP) gate sets, then the corresponding necessary and sufficient condition for a gauge transformation to preserve this constraint is that *M* be itself TP (that is, its first row should be (1, 0, 0,…).

This gauge freedom makes it difficult to compare two gate sets, since two apparently distinct gate sets may actually be equivalent. Most of the metrics used to measure distance between two gates are not gauge-invariant (for example, fidelity, trace-norm distance and diamond norm distance are all gauge-variant). So, while it would be ideal to work only with gauge-invariant metrics, we have very few metrics (and developing them and championing their adoption to the scientific community is beyond the scope of this work). Instead, to generate meaningful metrics, we gauge optimize gate sets to make them as ‘close’ as possible before computing metrics.

Given a gate set 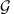 and a target 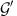, we transform 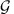 by *M* (as above) where *M* is chosen to optimize some criterion of ‘closeness’ between 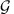 and 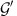. This is ‘gauge optimization’. The final output of GST is thus the gate set that is most similar to the target, according to some gauge-variant quantity, among a class of gauge-equivalent gate sets. In the work reported here, we minimize (for convenience) a weighted Frobenius distance:


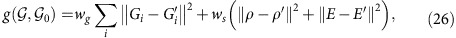


where 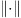 denotes the Frobenius norm, *G*_*i*_ ranges over all gates in the set, and *w*_*g*_ and *w*_*s*_ are weighting factors. The weight ratio *w*_*s*_/*w*_*g*_ allows us to fine-tune the relative contributions of discrepancies in logic gates and in SPAM. This is important because their respective natural uncertainties are usually quite different; gates can be probed far more accurately than SPAM. Thus, typically, 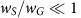; we weight the gate matrix elements more highly because they are known more precisely. We use an iterative numerical method to find an *M* that minimizes this quantity.

Each gate set is gauge-optimized as a whole; we report all metrics using gates in a single gauge. It would be incorrect to separately optimize the gauge for different reported quantities (for example, gauge-optimizing for the fidelity of a single gate and reporting each such best-fidelity separately). Finally, we note that the process of gauge optimization against a reference gate set is sufficient to solve gauge ambiguity issues. That is, any quantity of interest that is not inherently gauge-invariant (for example, diamond norm) becomes so when this gauge optimization is performed. This numerical optimization process is not physically elegant, but is adequate for the practical applications we consider here.

### Error bars

In interpreting the GST analyses (and in particular confirming the claim that we have demonstrated FT), it is necessary to assign error bars to gate set estimates (and derived quantities thereof). For most GST-derived quantities, we use Hessian-based likelihood ratio (LR) confidence regions, while for RB-related quantities, we use parametric and non-parametric bootstrapping. We also use parametric bootstrapped error bars as a sanity check on our Hessian-based LR confidence regions, and find them to be in good agreement. Unless otherwise stated, all error bars indicate ∼95% (2*σ*) confidence intervals.

We employ two flavours of bootstrapping: parametric and non-parametric. Both derive statistical quantities of interest from ensembles of simulated data sets, but these data sets are generated in different ways.

For the parametric bootstrap, ensembles of data sets are generated by first computing the GST estimate of the experimental data set in question, and then using this estimate to generate an ensemble of new data sets, each of which has the same experiments and number of shots per experiment as the actual experimental data set.

For the non-parametric bootstrap, ensembles of data sets are generated by simply resampling the experimental data set with replacement. In both parametric and non-parametric bootstraps, we typically generate an ensemble of 100 data sets, to ensure good statistics.

GST is used to map each resampled data set to a gate set estimate. Each gate set is gauge-optimized to match the experimental GST estimate as closely as possible. Then, from this ensemble of gauge-optimized gate sets, any statistical quantity of interest (such as s.d.) may be calculated for process matrix elements or for derived quantities such as diamond norm.

**Figure 8:**
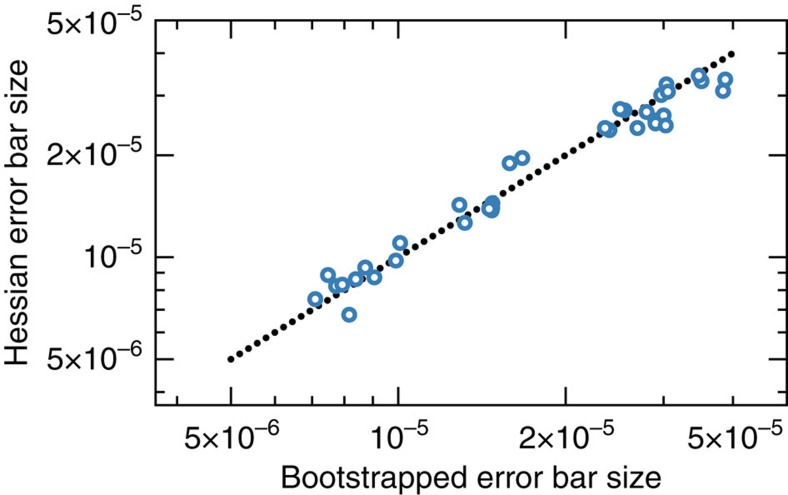
Comparison of error bar generation techniques. When computing error bars on GST estimates in this manuscript, we typically use LR confidence regions, computed using the Hessian of the log-likelihood function. However, another common approach is through parametric bootstrapping. Here we show a log–log scatter plot of error bars on gate elements from GST estimate of our data from 30 March 2015. The *x* axis corresponds to error bars calculated via parametric bootstrapping, whereas the *y* axis corresponds to likelihood ratio (LR) confidence regions computed using the Hessian of the log-likelihood function. The dotted line corresponds to *y*=*x*. Both methods are described in the methods section ‘Error Bars’. The strong correlation shown here demonstrates the consistency between parametric bootstrapping techniques and LR confidence regions.

We use bootstrapped error bars for two purposes. First, they serve as a sanity check on the more rigorous (but tricky to implement) LR confidence regions described in the following subsection. In Fig. 8, we compare the performance of parametric bootstrapping to the LR method, and see good agreement. Second, we use bootstrapping to put error bars on experimental RB decay rates. These are model-free and therefore not amenable to LR confidence regions. Error bars on experimental RB decay rate error bars were calculated via non-parametric bootstrapping, while error bars on simulated RB decay rates were calculated via parametric bootstrapping on the underlying GST estimate used to generate the RB data.

Bootstrapping is a very general method for generating error bars, but it is (1) not always reliable, (2) subject to small-sample errors unless very many Monte Carlo samples are generated and (3) quite time-consuming (up to 24 h of computer time were required to generate the 100 samples used for this paper). Likelihood ratio (LR) confidence regions^59^ are preferable in most ways, and we use them as our primary source for ‘error bars’.

The basic theory for LR confidence regions, as applied to quantum tomography, can be found in ref. 59. Confidence regions have a solid (if often misunderstood) statistical meaning: if an estimator generates confidence regions with a confidence level of 1−*α*, then with probability at least 1−*α* (taken over the ensemble of all possible data sets), the confidence region assigned by the estimator will contain the true parameter value. This does not mean ‘Given particular error bars, the probability that they contain the truth is 1−*α*,’ as there is no random variable to take a probability with respect to once the estimate has been assigned.

As implemented here, GST has two convenient properties. First, it yields a likelihood function that is well approximated by a Gaussian (because the total number of samples is quite large). Second, it involves no explicit constraints, meaning that the MLE is never squashed against a boundary (as it often is in standard state and process tomography, where the positivity constraint is critical to ensuring a physically valid estimate). These properties mean that we can approximate the log-likelihood function by a quadratic function, whose shape is given by the Hessian (matrix of second derivatives) of log 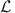 at the MLE. This Hessian defines a covariance tensor in gate set space, which (when scaled by an appropriate factor) defines an ellipsoid that is a valid 1−*α* confidence region.

Writing down this ellipsoid explicitly (as a covariance tensor) is possible, but not useful in practice. Instead, we use it to define error bars (confidence intervals) for all relevant scalar quantities (including fidelities, diamond norms, gate matrix elements and so on).

Let 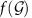 be a scalar function of a gate set. We define a 95% confidence interval around the best-estimate value of 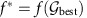 by computing


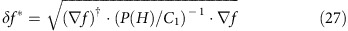


where *P*(*H*) is the Hessian projected onto the (local) space of non-gauge gate set parameters, and we have linearized 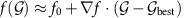. *C*_1_ is a scalar constant which satisfies CDF_1_(*C*_1_)=0.95, where CDF_1_ is the cumulative density function of the 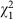 probability distribution. With *δf* so defined, f*±*δf* specifies the 95% confidence interval for *f*. Within the linear approximation to *f*, which is valid for small *δf*, this interval corresponds to minimizing and maximizing the value of *f* over the contour of the log-likelihood corresponding to a 95% confidence interval if the log-likelihood had a single parameter.

We emphasize that this does not construct a 95% confidence region. There are roughly 34 gauge-invariant parameters in a gate set; the threshold used here implies 95% confidence intervals for each of them. The resulting region contains the truth only if every one of the intervals contains its parameter, which occurs with probability at least 0.95^34^≈17%.

We believe this is a more meaningful way to report ‘error bars’ than to report a 95% confidence region for the entire gate set. For one thing, it is consistent with the error bars reported by the bootstrap (which yields standard errors for each parameter independently, and would have to be expanded significantly to represent a joint confidence region). Empirically, we find that definition 27 correlates closely with the 2*σ* error bars on gate elements computed by parametric bootstrapping (see Fig. 8). Furthermore, we use the confidence region primarily to report uncertainties on single quantities (for example, diamond norms), independent of the others.

### Data availability

The GST and RB analysis in this paper was performed using the open-source software pyGSTi (python GST implementation)^60^, which was developed for this work. All data sets and analysis scripts necessary to reproduce the results presented here are available online as supplemental information at https://doi.org/10.5281/zenodo.231329.

## Additional information

**How to cite this article:** Blume-Kohout, R. *et al*. Demonstration of qubit operations below a rigorous fault tolerance threshold with gate set tomography. *Nat. Commun.* **8,** 14485 doi: 10.1038/ncomms14485 (2017).

**Publisher’s note**: Springer Nature remains neutral with regard to jurisdictional claims in published maps and institutional affiliations.
