# Publisher Correction: Demonstration of qubit operations below a rigorous fault tolerance threshold with gate set tomography

**DOI:** 10.1038/ncomms16226

**Published:** 2018-06-25

**Authors:** Robin Blume-Kohout, John King Gamble, Erik Nielsen, Kenneth Rudinger, Jonathan Mizrahi, Kevin Fortier, Peter Maunz

Nature Communications 8: Article number: 14485; DOI: 10.1038/ncomms14485 (2017); Published: 02 15 2017; Updated: 06 25 2018

The original HTML version of this Article omitted the article number; it should have been ‘14485’. This has now been corrected in the HTML version of the Article. The PDF version was correct from the time of publication.

